# Rice straw-derived smoke water promotes rice root growth under phosphorus deficiency by modulating oxidative stress and photosynthetic gene expression

**DOI:** 10.1038/s41598-023-41987-5

**Published:** 2023-09-08

**Authors:** Sompop Pinit, Lalichat Ariyakulkiat, Juthamas Chaiwanon

**Affiliations:** 1 Center of Excellence in Environment and Plant Physiology, Department of Botany, Faculty of Science, Chulalongkorn University, Bangkok, Thailand; 2 Department of Biochemistry, Faculty of Medical Science, Naresuan University, Phitsanulok, Thailand; 3 Center of Excellence in Medical Biotechnology, Faculty of Medical Science, Naresuan University, Phitsanulok, Thailand

**Keywords:** Plant development, Abiotic

## Abstract

Plant-derived smoke has been shown to promote plant growth and seed germination, but its roles and mechanisms in response to nutrient deficiency stress remain unclear. Plants respond to phosphorus (P) deficiency by undergoing morphological, physiological, and transcriptional changes in order to improve nutrient uptake efficiency. Here, we showed that rice straw-derived smoke water could promote root growth in rice (*Oryza sativa* cv. Nipponbare) grown under P-sufficient and P-deficient conditions. Transcriptome analysis of the root tissues identified 1309 genes up-regulated and 1311 genes down-regulated by smoke water under P-deficient conditions. The GO terms ‘glutathione transferase activity’ and ‘photosynthesis—light reaction’ were found to be significantly enriched among the genes that were up- and down-regulated by smoke water, respectively. Biochemical analysis showed that smoke water reduced P-deficient-induced accumulation of H_2_O_2_ and malondialdehyde (MDA), a lipid peroxidation marker, reduced sucrose contents, but increased Fe accumulation. Furthermore, smoke water suppressed the expression of strigolactone biosynthesis genes, which were strongly induced by P deficiency as an adaptive strategy to improve root P uptake. These results revealed a potential mechanism by which smoke water promotes root growth and interacts with P deficiency-induced transcriptional regulation to mitigate P deficiency stress in rice.

## Introduction

Biomass burning in open fields is a common agricultural practice in several areas, particularly in Asia^1^. This burning activity typically takes place before and after cultivation as a quick and easy way of eliminating previous crop residues and weeds. Although fire and smoke have been shown to promote germination, especially for fire-prone plant species, such practices not only cause air pollution but also kill beneficial soil microorganisms^2^. Recently, plant-derived smoke and aqueous extracts obtained from smoke have received a lot of attention in agriculture and horticulture due to their potent effect similar to that of plant growth regulators^3^.

Smoke contains a number of different compounds, including biologically active compounds with potential agricultural use such as karrikins (KARs) and butenolides^4,5^. Smoke water has been reported to promote seed germination and growth in several plant species, including Arabidopsis, cucumber, scotch marigold, and rice^6,7^. However, the mechanisms by which smoke water promotes plant growth and its interaction with nutrient deficiency stress are not fully understood.

Phosphorus (P) is an essential macronutrient that plays a crucial role in energy metabolism and plant growth and development. However, P is often bound to soil particles and is relatively immobile, making it unavailable to plants even when P is present in the soil^8^. Plants adapt to P availability by undergoing morphological, physiological, and transcriptional changes in order to improve nutrient uptake efficiency^9^. Modification of root system architecture in response to P deficiency is one of the strategies to improve P acquisition^10^. In rice (*Oryza sativa* L.), the ability of roots to elongate under P deficiency varied greatly among rice populations^11,12^. Previous studies have shown that a gene encoding the protein kinase Pstol1 (Phosphorus-starvation tolerance 1), which is located in a major quantitative trait locus for P-deficiency tolerance, *Pup1*, is responsible for enhanced root growth and large root system, contributing to increased nutrient uptake and P-deficiency tolerance^13^. Overexpression of the gene could promote root growth and improve P-deficiency tolerance in rice cv. Nipponbare, whose genome lacks *PSTOL1* and does not exhibit P deficiency-induced root elongation traits^11,13^.

In this study, the effects of rice straw-derived smoke water treatment on root growth of Nipponbare rice seedlings under P-sufficient and P-deficient conditions were investigated in a hydroponic system. Transcriptomic analysis of root tissues treated with smoke water revealed that smoke water altered the expression of genes involved in oxidative stress, photosynthesis, Fe deficiency, and strigolactone biosynthesis. The accumulation of H_2_O_2_, an oxidative stress marker, sucrose, and P and Fe in root tissues were also determined to investigate the mechanism of smoke water in promoting rice root growth and mitigating P deficiency stress.

## Results

### Smoke water promotes rice root growth

**Figure 1:**
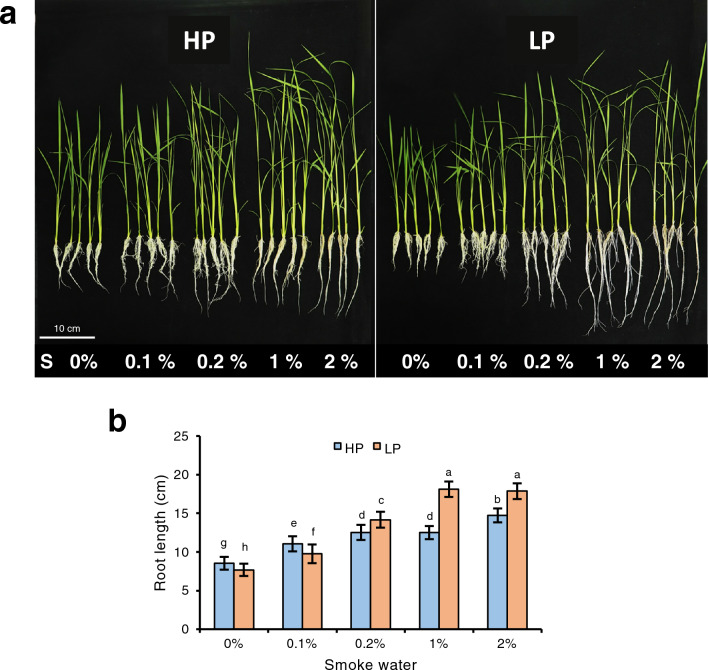
Smoke water enhanced P deficiency-induced root elongation in rice. (**a**) Rice seedlings (cv. Nipponbare) were grown hydroponically in P-sufficient (HP) or deficient (LP) conditions and treated with various concentrations of smoke water in the nutrient solutions. Scale bar, 10 cm. (**b**) Quantification of root lengths. Data are means ± s.d. (n = 4 biological replicates with 6 plants each). Different letters indicate significant differences (*p* < 0.05) according to Duncan’s multiple range test (DMRT).

Rice seedlings (cv. Nipponbare) were grown hydroponically in P-sufficient (HP) or deficient (LP) conditions and treated with various concentrations of smoke water in the nutrient solutions. Nipponbare root growth was slightly inhibited under P deficiency, consistent with previous reports^11^ (Fig. 1). Smoke water treatments promoted shoot and root growth in a dose-dependent manner under both HP and LP conditions. When 0.2%, 1% or 2% smoke water was applied, root elongation could be promoted under LP condition at higher levels than under HP condition (Fig. 1). This suggests that smoke water could enhance P deficiency-induced root elongation in Nipponbare.

### Transcriptome profiling of smoke water-treated rice roots

To understand the mechanisms by which smoke water promotes root elongation and response to P deficiency, we performed RNA-seq analysis of root tissues derived from rice seedlings treated with or without 1% smoke water (+S or −S) under HP or LP conditions for 3 days. Each treatment included three biological replicates. mRNA libraries were sequenced using the Illumina NovaSeq platform. After pre-processing, filtering low-quality reads, and alignment to the RAP-DB reference genome (IRGSP 1.0.21), comparison of expression profiles was performed using DESeq2^14^. Genes that were significantly differentially expressed by more than 2 folds (|log_2_fold change| > 1 and adjusted p-value < 0.05) were included in the differentially expressed gene (DEG) list for further analysis.

**Figure 2:**
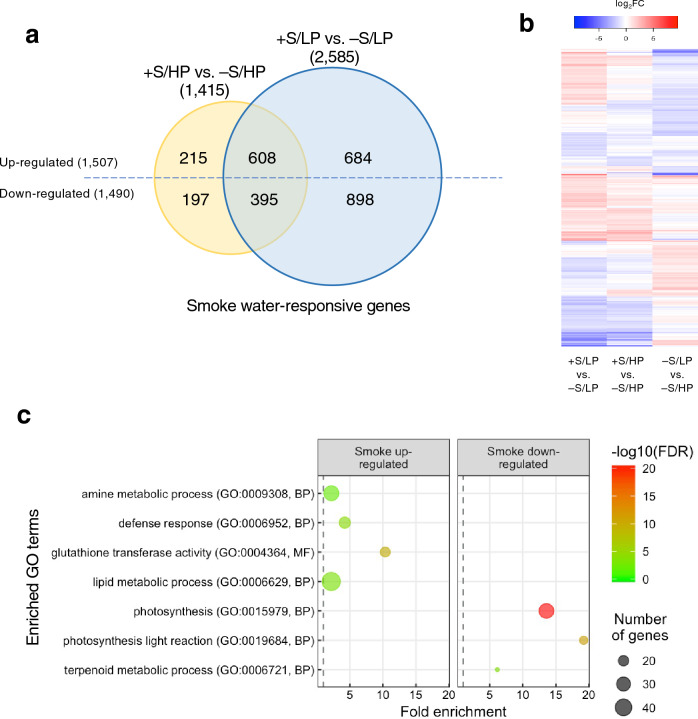
Transcriptome analysis of roots treated with smoke water under P-sufficient and -deficient conditions. Root tissues were harvested from 10-day-old rice seedlings grown hydroponically in Pi-sufficient (HP) or deficient (LP) conditions and treated with or without 1% smoke water (+S or −S) for 3 days. (**a**) Venn diagram showing the overlap between the lists of smoke water up- and down-regulated genes (|log_2_fold change| > 1; adjusted p-value < 0.05) in HP and LP conditions. (**b**) Hierarchically clustered heatmap displaying the log_2_FC values of all smoke water-responsive or P-deficiency-responsive genes. (**c**) GO biological process (BP) and molecular function (MF) term enrichment analysis of the smoke water up- and down-regulated gene lists.

To identify smoke water-responsive genes in HP condition, the treatment +S/HP was compared with −S/HP; 841 and 609 genes were up- and down-regulated by smoke water treatment, respectively. In LP condition (+S/LP vs. −S/LP), smoke water up-regulated 1309 genes and down-regulated 1311 genes, with 608 and 395 genes also up- and down-regulated, respectively, by smoke water in HP condition. Among all smoke water-responsive genes, there were 35 genes that showed opposite regulation by smoke water under HP and LP conditions (up-regulated in one and down-regulated in the other) and were excluded from the Venn diagram (Fig. 2a, Supplementary Table S1).

To identify P deficiency-responsive genes, the treatment −S/LP was compared with −S/HP; 969 and 700 genes were up- and down-regulated by P deficiency, respectively. Expression heatmap analysis of 4281 genes, which were significantly differentially expressed in at least one of the comparisons (+S/LP vs. −S/LP, +S/HP vs. −S/HP, and −S/LP vs. −S/HP), showed that the effect of smoke water on gene expression is quite distinct from the effect of P deficiency on root transcriptomes (Fig. 2b). In addition, the effects of smoke water treatment on root transcriptomes under both LP and HP conditions were similar, but the degrees of expression fold changes under LP condition were higher.

### Gene ontology enrichment analysis

To investigate the effects of smoke water on biological processes and molecular functions, we performed gene ontology (GO) enrichment analysis of the lists of smoke water-responsive genes under LP condition (Fig. 2c). GO terms significantly enriched for the smoke water-up-regulated gene list included glutathione transferase activity (GO:0004364), defense response (GO:0006952), amine metabolic process (GO:0009308), and lipid metabolic process (GO:0006629). GO terms significantly enriched for the smoke water-down-regulated gene list included photosynthesis (GO:0015979), photosynthesis—light reaction (GO:0019684), and terpenoid metabolic process (GO:0006721).

### Oxidative stress-related genes

**Figure 3:**
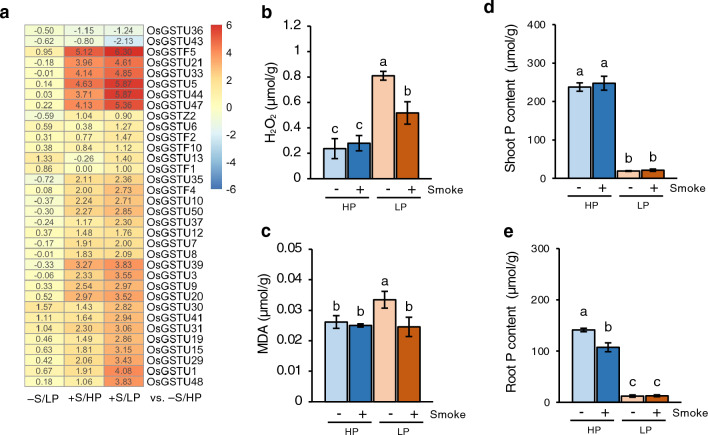
Smoke water promoted expression of genes encoding glutathione-S-transferases (GSTs) and reduced accumulation of H_2_O_2_ and oxidative stress markers in P-deficient roots. (**a**) Heatmap represents log_2_FC values of genes in the −S/LP, +S/HP or +S/LP compared with −S/HP conditions. Only genes that showed statistical significance in at least one of the comparisons were included in this figure. (**b**,**c**) Quantification of H_2_O_2_ (**b**) and malondialdehyde (MDA) (**c**) in P-deficient roots. (**d**,**e**) Total P contents in shoots (**d**) and roots (**e**) of rice seedlings. Data are means ± s.d (n = 3 biological replicates). Different letters indicate significant differences (*p* < 0.05) according to Duncan’s multiple range test (DMRT).

The term ‘glutathione transferase activity’ was enriched among the smoke water-up-regulated genes with 10.35 fold enrichment. A number of genes encoding glutathione-s-transferases (GSTs) were upregulated by smoke water in both HP and LP conditions. Compared to the control (−S/HP), the expression levels were strongly upregulated by smoke in LP more than in HP (Fig. 3a, Supplementary Table S1). We identified 26 *OsGSTUs*, five *OsGSTFs* and one *OsGSTZ* up-regulated by smoke water, with the exception of *OsGSTU36* and *OsGSTU43* that were significantly down-regulated.

GSTs play important roles in the detoxifying mechanisms in response to abiotic stresses and stress tolerance in plants^15^. Previous studies have shown that P deficiency, as well as other abiotic stresses, induced the accumulation of H_2_O_2_ and malondialdehyde (MDA), a lipid peroxidation marker caused by oxidative stress^16^. Quantification of H_2_O_2_ and MDA levels in the roots showed that P-deficient roots accumulated higher levels of H_2_O_2_ and MDA and that smoke water treatment could remarkably reduce both H_2_O_2_ and MDA (Fig. 3b,c). This suggests that smoke water treatment is able to reduce P deficiency-induced oxidative stress, potentially by upregulating the expression of GSTs.

To test whether smoke water treatments mitigate P deficiency-induced stress by improving plant P status, total P contents in shoots and roots were determined. LP treatments dramatically reduced P contents in both shoots and roots, whereas smoke water treatments did not significantly increase P contents when compared to the control of the same P condition (Fig. 3d,e). This illustrated that both the −S/LP and +S/LP samples were similarly P-deficient. Phosphate (Pi) starvation-induced (PSI) genes, including Purple Acid Phosphatases (*PAPs*), Pi Transporters (*PTs*), *OsSPX,* and *OsSQD2* genes, were also upregulated at similar levels in both −S/LP and +S/LP samples (Supplementary Fig. S1). Taken together, these results suggest that smoke water did not alter degrees of P deficiency as reflected by total P contents and transcript levels of most genes involved in Pi starvation responses but could mitigate P-deficiency stress.

### Photosynthesis-related genes

**Figure 4:**
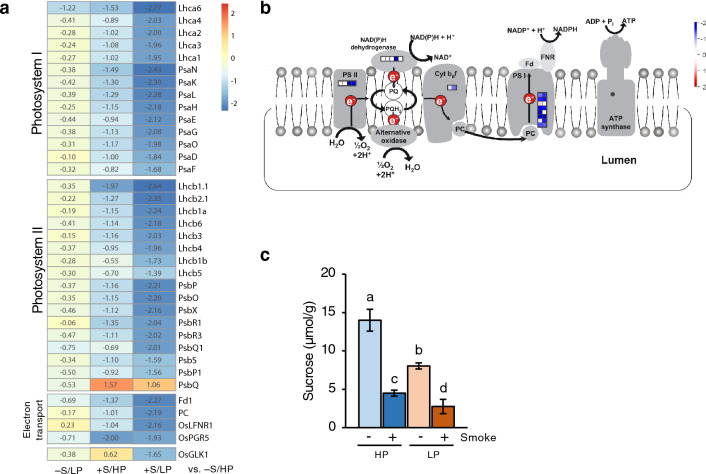
Smoke water repressed expression of genes involved in photosynthesis and reduced sucrose accumulation in roots. (**a**) Heatmap represents log_2_FC values of genes in the −S/LP, +S/HP or +S/LP compared with −S/HP conditions. Only genes that showed statistical significance in at least one of the comparisons were included in this figure. (**b**) MapMan overview of the genes functioning at the thylakoid membrane of the chloroplast. The individual genes are exhibited by small color squares, showing log_2_FC values of genes in the +S/LP vs. −S/LP comparison. (**c**) Sucrose contents in the roots. Data are means ± s.d. (n = 3 biological replicates). Different letters indicate significant differences (*p* < 0.05) according to Duncan’s multiple range test (DMRT).

The GO biological processes ‘photosynthesis’ and ‘photosynthesis—light reaction’ were significantly enriched in the smoke water-down-regulated gene list with 13.59 and 19.22 fold enrichment, respectively (Fig. 2c). Smoke water significantly down-regulated 5 genes encoding photosystem I (PSI) light-harvesting chlorophyll *a/b*-binding (LHCA) proteins, 8 genes encoding photosystem II (PSII) light-harvesting chlorophyll *a/b*-binding (LHCB) proteins, 9 genes encoding PSI reaction center subunits (PsaD, PsaE, PsaF, PsaG, PsaH, PsaK, PsaL, PsaN, and PsaO), and 8 genes encoding PSII components (Fig. 4a,b, Supplementary Table S1). In addition to the genes encoding PSI and PSII components, Ferredoxin 1 (Fd1), Plastocyanin (PC), and leaf-type ferredoxin-NADP^+^-oxidoreductase 1 (LFNR1) that are involved in electron transport, as well as OsPGR5 (Proton Gradient Regulation 5), which is required for PSI cyclic electron transport, were also down-regulated by smoke water. Moreover, smoke water treatment in the P-deficient condition also down-regulated the MYB transcription factor OsGLK1 (Golden 2-like 1), a major regulator of chloroplast development^17^. Measurement of sucrose contents in root tissues showed that P deficiency decreased sucrose contents by 42.57% in the absence of smoke water, and smoke water decreased sucrose contents by 67.89% and 65.69%, when compared to the control in HP and LP, respectively (Fig. 4c). Although root chloroplasts are capable of photosynthesis, their efficiency is much lower than that of shoots^18^. This finding suggests that, rather than having a negative impact directly on root carbon assimilation, smoke water may affect light-induced ROS production in the root chloroplasts, and shoot–root translocation or sink strength of the roots.

### Fe deficiency-related genes

The antagonistic interaction between P and Fe has been well established^19–21^. Under Fe deficiency, rice employs both reduction and chelation strategies to increase Fe acquisition and transports. In rice, Fe-deficiency-responsive genes include genes encoding ferric-chelate reductase (FRO), ferrous transporters (IRTs), deoxymugineic acid (DMA) biosynthesis enzymes, Fe (III)-DMA transporters (Yellow Stripe family transporters, YSLs), as well as transcription factors, OsIRO2 and OsIRO3, which are responsible for controlling the DMA synthesis and Fe transporter genes^22,23^.

**Figure 5:**
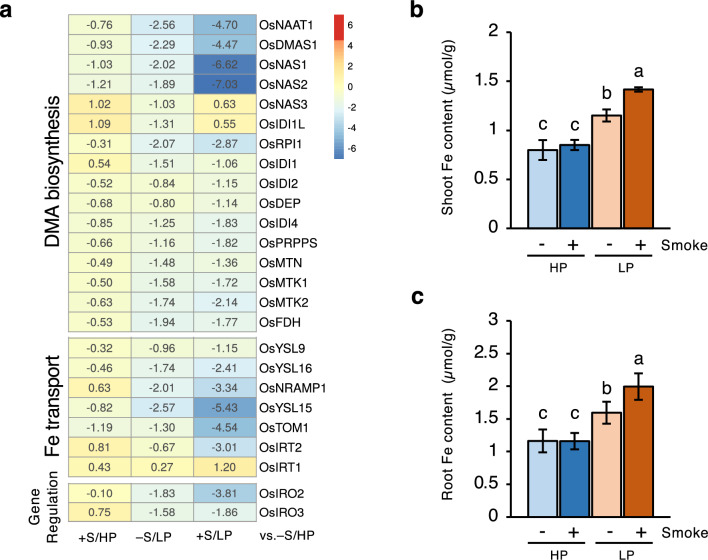
Smoke water increased Fe contents and repressed Fe deficiency-related gene expression in P-deficient plants. (**a**) Heatmap represents log_2_FC values of genes in the −S/LP, +S/HP or +S/LP compared with −S/HP conditions. Only genes that showed statistical significance in at least one of the comparisons were included in this figure. (**b**,**c**) Fe contents in shoots (**b**) and roots (**c**) of rice seedlings. Data are means ± s.d. (n = 3 biological replicates). Different letters indicate significant differences (*p* < 0.05) according to Duncan’s multiple range test (DMRT).

In the absence of smoke water treatment, P deficiency down-regulated expression of genes involved in Fe homeostasis and increased Fe concentrations in shoots and roots (Fig. 5), consistent with previous reports^20^. This is partly due to increased availability of Fe in the Pi-deficient media^19^. Interestingly, smoke water treatment further enhanced suppression of the Fe-related genes under P deficiency. This expression data was correlated with the elevated Fe concentrations in the smoke water-treated Pi-deficient plants (Fig. 5). It is possible that smoke water could further increase Fe availability in the Pi-deficient media and/or increase Fe accumulation in plants due to the larger root system (Fig. 1).

### Strigolactone-related genes

**Figure 6:**
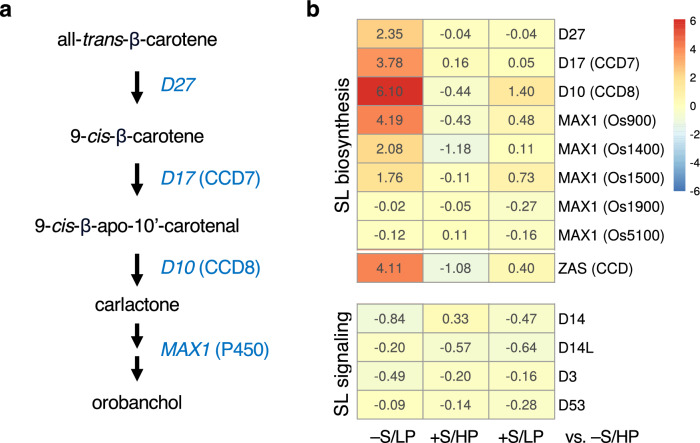
Smoke water suppressed expression of Pi deficiency-induced strigolactone biosynthesis genes. (**a**) Strigolactone biosynthesis pathway. (**b**) Heatmap represents log_2_FC values of genes in the −S/LP, +S/HP or +S/LP compared with −S/HP conditions.

Strigolactone (SL) plays key roles in plant adaptation to nutrient deficiency by promoting arbuscular mycorrhiza (AM) colonization in order to increase nutrient foraging. In the absence of smoke water, P deficiency strongly induced expression of SL biosynthesis enzymes, but not SL signaling components, consistent with previous findings^24^. Smoke water treatment in HP condition did not alter expression of SL biosynthesis genes (Fig. 6). Interestingly, the P deficiency-induced SL biosynthesis gene expression was strongly suppressed in the presence of smoke water (Fig. 6). Moreover, smoke water also suppressed the upregulation of *ZAS* in response to P deficiency. The gene *ZAS* encodes a carotenoid cleavage dioxygenase (CCD) enzyme, zaxinone synthase, which produces zaxinone, an apocarotenoid metabolite that regulates growth and SL biosynthesis in rice^25^. These findings suggest that smoke water could alter the transcriptional control of these genes and affect the Pi starvation-induced mechanism. It also suggests that smoke water could alter the metabolic balance of these carotenoid-derived signaling molecules under P deficiency.

## Discussion

**Figure 7:**
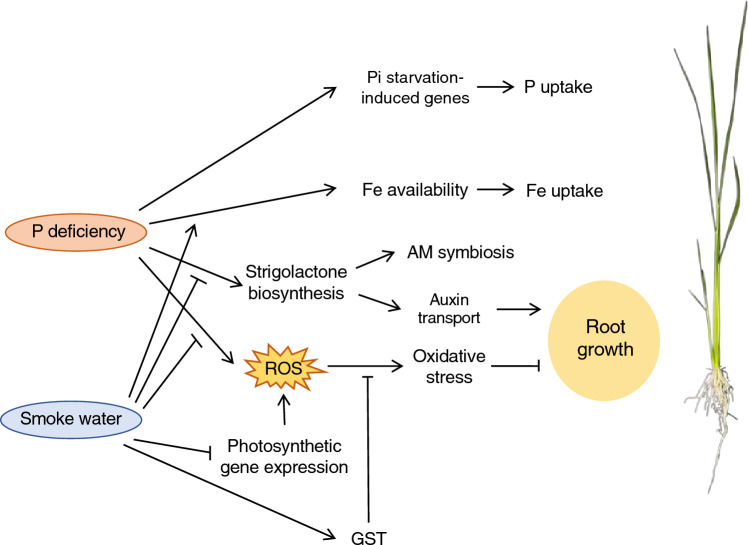
A proposed schematic model of smoke water regulating rice root elongation and P deficiency responses.

P deficiency has been shown to alter root system architecture to enhance P foraging and uptake in several species^9,26^. In rice, the effect of P deficiency on root system development depends on underlying genetic control^11,27^. Here, we demonstrated the growth-promoting effects of rice straw-derived smoke water on Nipponbare rice root under P-deficient condition, in which root growth was inhibited in the absence of smoke water treatment (Fig. 1). This suggests that smoke water could promote root elongation under P deficiency through mechanisms independent of *PSTOL1*, which functions to enhance root system but is absent from the Nipponbare genome^13^. Our transcriptome analysis revealed that smoke water modulated the expression of genes involved in oxidative stress, photosynthesis, and SL biosynthesis (Fig. 2). Based on the results, we proposed a schematic model of smoke water regulating rice root elongation and P deficiency responses (Fig. 7).

The antioxidative role of glutathione in reducing oxidative stress and preventing lipid peroxidation has been well documented^28^. In this study, smoke water strongly induced expression of GSTs, and the degree of induction was increased by P deficiency (Fig. 3a). Smoke water treatment also reduced H_2_O_2_ and MDA levels in P-deficient roots (Fig. 3b,c). These results suggest that smoke water may alleviate oxidative stress on roots induced by P deficiency^29^, consequently contributing to root elongation under P deficiency. A recent proteomic analysis has demonstrated that smoke water induced glutathione-related proteins and improved shoot growth in maize seedlings^30^. Glutathione also modulates auxin homeostasis to regulate root system adaptation to P deficiency in Arabidopsis^31^. Furthermore, it has been shown in wheat that combining P and Fe deficiency could promote expression of GSTs and recover root elongation, which was otherwise inhibited under Fe deficiency^32^. Wang et al. reported consistently that catechol, a major component of smoke, upregulated a number of genes related to redox homeostasis, leading to smoke-induced root cell elongation through spatial changes in redox status in *Nicotiana attenuata* roots^33^. Previous research has demonstrated that ROS controls the balance between cell proliferation and differentiation in roots, where O_2_^**·**−^ accumulates in the meristem and H_2_O_2_ accumulates in the elongation zone, thereby determining meristem size and root length^34^. Further research into tissue-specific ROS distribution in root tips using histochemical assays would provide a better understanding of the effects of smoke water on root cell proliferation and differentiation.

Our transcriptome analysis showed that smoke water treatment repressed expression of photosynthetic genes, particularly those involved in the light reaction at the thylakoid membrane, and the repression was stronger under P deficiency (Fig. 4). It should be noted that, in our study, roots were grown under in vitro conditions with some exposure to light, which may contribute to the responses of the photosynthetic genes. P deficiency has been shown to reduce photosynthesis in shoots as well as the expression of photosynthetic genes in roots of Arabidopsis, soybean, and wheat^35–38^. Loss-of-function mutations of a gene encoding tyrosylprotein sulfotransferase (TPST) and overexpression of GLK transcription factors in Arabidopsis resulted in ectopically activated photosynthesis in the roots, increased light-induced ROS production in root cells, and hypersensitive root growth inhibition under P deficiency^37^. Although the biological significance of photosynthesis in roots remains unclear, this evidence demonstrates that suppression of photosynthetic gene expression in roots is required for sustained root growth in vitro under P deficiency^37^. Hence, smoke water application may promote rice root growth by suppressing the expression of photosynthetic genes, which may contribute to light-induced ROS production. Expression of photosynthetic genes is controlled by several transcription factors, including HY5, PIFs, GLKs, GNC, and GNL^39–41^. Only *OsGLK1* was found to be significantly differentially expressed by smoke water in our transcriptome analysis. Hydroquinone, a bioactive compound in plant-derived smoke^42^, has been shown to inhibit transcription of photosynthetic genes in marine diatoms^43^. The bioactive compounds in smoke water that are responsible for the transcriptional regulation of photosynthetic genes will need to be identified through additional research.

Sucrose not only functions as a source of energy for cellular metabolism, but also acts as a signaling molecule in multiple pathways to coordinate the growth of above-ground shoots with the underground roots^44^. P deficiency has been reported to increase sugar accumulation in bean, soybean, and rice roots, decrease sugar accumulation in cucumber roots, and remain unchanged in Arabidopsis roots^44–46^. In our observation, sucrose contents were reduced in P-deficient and smoke water-treated roots (Fig. 4c). Previous studies have shown that the effects of P deficiency on root growth vary depending on plant genotype, growth conditions (soil, hydroponic, or agar), light conditions, and root illumination^11,47–49^. These factors may explain the variation in sugar contents observed across studies. Interestingly, despite the reduced sucrose content, our results showed that smoke water promoted root growth, suggesting that smoke water may promote metabolic efficiency. P deficiency has been shown to induce lysigenous aerenchyma formation in some genotypes of rice, maize, and common bean. This adaptive trait contributes to decreased respiration and increased P utilization efficiency, thus increasing metabolic efficiency and allowing plants to develop additional root structures, such as longer roots, lateral roots, and root hairs, to explore the soil^50,51^. Further study is needed to determine whether smoke water can promote aerenchyma formation and improve metabolic efficiency in P-deficient conditions.

Our findings demonstrate P deficiency-induced transcriptomic changes, including upregulation of the PSI genes, downregulation of Fe homeostasis genes, and upregulation of SL biosynthesis genes, which are consistent with previous reports^19,32^. Under P deficiency, smoke water treatment did not alter the expression of the PSI genes but enhanced the suppression of Fe homeostasis genes. It is possible that smoke water may affect P acquisition efficiency in soil-grown plants, potentially by promoting root growth and improving foraging capacity. However, in this study, the smoke water-treated P-deficient plants could not benefit from the larger root system because the amount of available Pi in hydroponic conditions was limited and finite. Further studies are needed to study the effects of smoke water on root growth and P uptake in the soil. In contrast, Fe uptake was enhanced by smoke water under P deficiency, which could be attributed to the increase of Fe availability in the Pi-deficient nutrient solution^19^, as well as the larger root system of the smoke water-treated plants.

Pi and nitrate deficiencies have been shown to induce strigolactone biosynthesis, which plays crucial roles in regulating root adaptation by modulating auxin transport^24^. Moreover, SLs in root exudates not only promote symbiosis with arbuscular mycorrhiza (AM) to increase nutrient uptake, but also trigger seed germination of parasitic weeds^52,53^. Our results revealed that smoke water treatment inhibit the expression of SL biosynthesis genes, which were strongly induced in P-deficient plants. This suggests that smoke water interacts with the Pi starvation-induced transcriptional control and may reduce levels of SLs exuded by the roots, which could consequently affect mycorrhization. On the other hand, it is also possible that smoke water may contain compounds that have similar properties as SLs, and thus the presence of such compounds could feedback regulate endogenous SL biosynthesis under P deficiency^54^. In this case, the chemicals may have the potential to induce germination of root parasitic weeds before farming, an agricultural technique known as suicidal germination^55^. Indeed, karrikins, which are plant growth regulators derived from plant material smoke, have structural similarities to strigolactones and function via a signaling pathway closely related to that of SL^5^. Further experiments are needed to determine whether smoke water reduces SL levels in plants and root exudates, how that might affect SL function in plant growth and development, including Pi starvation responses and AM colonization, and whether smoke water could affect germination of the parasitic weeds.

## Conclusion

Smoke water has been used in agriculture in various ways, including as a seed treatment, foliar spray, or soil amendment, and several studies have shown positive effects of smoke water on seed germination and plant growth. However, the effects of smoke water on plant growth and development could vary depending on the type of plant and the concentration and composition of smoke water^3,7,56^. In this study, we showed the promoting effects of rice straw-derived smoke water on root growth of P-deficient rice and provided further understandings of potential mechanisms through transcriptional modulation of genes involved in oxidative stress, photosynthesis, and SL biosynthesis. Further research is needed to fully understand its potential as a plant growth promoter across different plant species and under different environmental conditions.

## Materials and methods

### Production of plant-derived smoke water solution

Rice straw-derived smoke water was prepared using the method adapted from a previous study^7^. In brief, one kilogram of dried rice straws was burned in a closed burning system connected to a vacuum to draw the smoke through 1-L water in the bottle, in which the water-soluble compounds in the smoke could dissolve. The smoke–water solution was then used in the subsequent experiments.

### Plant materials and growth condition

Seeds of rice (*Oryza sativa* L.) cv. Nipponbare were obtained from the Center of Excellence in Environment and Plant Physiology, Chulalongkorn University, as used in the previous study^57^. Seeds were surface-sterilized and pre-cultured in water for 2 days, followed by half-strength Yoshida’s solution^58^ for 2 days under dark conditions. Then, the seedlings were grown in full-strength Yoshida’s solution under light conditions for 4 days before removing the seed endosperm and being transferred to P-sufficient (320 µM NaH_2_PO_4_, HP) and P-deficient (0.8 µM NaH_2_PO_4_, LP) conditions in the presence of various concentrations of smoke water. The seedlings were treated for 8 days for growth and biochemical analyses or 3 days for RNA extraction. The nutrient solutions were adjusted to pH 5.8 and renewed every 2 days. The seedlings were grown in a growth room at 30 °C with a 12 h/12 h light–dark cycle.

### RNA extraction, cDNA library construction and RNA-Seq

Whole root tissues were collected from treated plants and immediately frozen in liquid nitrogen. Three biological replicates, each consisting of 6 plants, were used for each treatment. Total RNA was extracted from the root tissues using PureLink RNA Mini Kit based on the manufacturer’s instructions (Invitrogen, USA) and genomic DNA was removed using DNase I (Thermo Fisher Scientific, USA). The quality of the total RNA was assessed by the Agilent 2100 Bioanalyzer (Agilent Technologies, USA), and all samples had an RNA integrity number (RIN) greater than 8.9. Purification of mRNA, library construction, and sequencing were carried out by Apical Scientific Sdn. Bhd. (Malaysia) using the Illumina NovaSeq 6000 sequencer (Illumina, USA) to obtain 2 × 150 bp paired-end reads according to the manufacturer’s instructions. The RNA-seq raw data were deposited in the Sequence Read Archive of the National Center for Biotechnology Information under accession number PRJNA924970.

### Data processing and bioinformatics analysis

Following pre-processing and filtering of low-quality reads, paired-end clean reads were aligned to the RAP-DB reference genome (IRGSP 1.0.21) using HISAT2 (version 2.1.0)^59^. Reads were assembled into transcripts using StringTie (version 2.0.6)^60^. Differential gene expression was performed using DESeq2 (version 1.31.6)^14^ and genes with an adjusted p-value < 0.05 and |fold change| > 2 (equivalent to |shrunken log_2_fold change| > 1) were considered to be significantly differentially expressed. Hierarchical clustering and heatmap analysis were performed using ‘hclust’ and ‘heatmap.2’ packages in R (version 3.5.3). Heatmaps showing expression levels of selected genes were plotted using ClustVis web tools^61^.

To investigate the effects of smoke water on biological processes and molecular functions, we performed gene ontology (GO) enrichment analysis of the list of genes responsive to smoke water under P-deficient conditions using agriGo v2.0 with Fisher’s exact test, Yekutieli (FDR under dependency < 0.05)^62^. Lists of the enriched GO terms were then summarized by REViGO^63^. Terms with FDR < 0.005 and fold enrichment > 2 were plotted using R script^64^. The log_2_FC values of genes in the +S/LP vs. −S/LP comparison were also imported into MapMan software^65^ to visualize genes functioning at the thylakoid membrane of the chloroplast.

### Measurement of P and Fe contents

Root and shoot samples were dried at 70 °C until a constant weight was recorded. To determine total P and Fe contents, dry root and shoot samples were digested with concentrated HNO_3_ using microwave digestion (UltraWAVE, Milestone, CT, United States). P contents were determined by the ammonium molybdate colorimetric assay^66^. In brief, diluted supernatants were mixed with molybdate blue reagent (0.4% w/v ammonium molybdate in 0.5 M H_2_SO_4_ and 10% w/v ascorbic acid; 6:1 ratio) and then incubated at 40 °C for 20 min. The absorbance was read at 820 nm, and the P content was calculated by comparing with a standard curve. Fe contents in the supernatant were determined using atomic absorption spectroscopy (Analytik Jena ZEEnit 700P, Germany).

### Measurement of H_2_O_2_ and MDA

Frozen tissue was ground and extracted with 0.1% trichloroacetic acid (TCA) on ice, and the supernatant was collected for H_2_O_2_ and MDA measurements. H_2_O_2_ was detected by mixing 0.5 ml of the supernatant with 0.5 ml of a 10 mM potassium phosphate buffer (pH 7.0) and 1 ml of 1 M potassium iodide, and measuring the absorbance at 390 nm. The H_2_O_2_ content was calculated using a standard curve^67^. MDA was detected by mixing 0.5 ml of the supernatant with 1 ml of a 0.5% thiobarbituric acid (TBA) solution, incubating the reaction mixture in boiling water for 30 min, and measuring the absorbance at 532 nm for the TBA-MDA complex and at 600 nm for non-specific turbidity correction. The MDA content was calculated using A_532_, A_600_ and the molar extinction coefficient of the MDA (115 mM^−1^ cm^−1^)^68^.

### Measurement of sucrose content

Sucrose content was extracted and measured following the method used in the previous study^69^. In brief, sucrose in ground frozen tissue was extracted with 80% ethanol by incubating at 80 °C for 40 min. The supernatant was collected, and the extraction process was repeated twice. The supernatant was then mixed with 0.2 ml of 2 M NaOH and heated at 100 °C for 5 min. The reaction mixture was added to 2.8 ml 30% HCl and 0.8 ml 0.1% resorcinol in 95% ethanol, and then incubated at 80 °C for 10 min. After the reaction was stopped, the absorbance was read at 480 nm, and the sucrose content was calculated using a standard curve.

### Statistical analysis

For quantitation of root length, the experiments included 4 biological replicates, each consisting of 6 plants. For measurement of P, Fe, H_2_O_2_, MDA, and sucrose contents, the experiments were performed with 3 biological replicates, each consisting of 8 plants. Means and standard deviations (SD) were calculated and analyzed by one-way analysis of variance (ANOVA). Mean comparison was calculated according to Duncan’s multiple range test (DMRT) using IBM SPSS Statistics 20.

### Ethical approval

Experimental research on plants in this study, including the collection of plant material, complies with relevant institutional, national, and international guidelines and legislation.

### Supplementary Information

Supplementary Information.

Supplementary Table S1.

## Data Availability

The RNA-seq data for all samples are available at the Sequence Read Archive of the National Center for Biotechnology Information under accession number PRJNA924970. All other data generated or analysed during this study are included in this published article.
