# Supplementary material for: Rice straw-derived smoke water promotes rice root growth under phosphorus deficiency by modulating oxidative stress and photosynthetic gene expression
Source: Sci Rep. 2023 Sep 8;13:14802. doi: 10.1038/s41598-023-41987-5 (PMC10491667; doi:10.1038/s41598-023-41987-5)
Supplement: Supplementary file 1 — Supplementary Information. [file 41598_2023_41987_MOESM1_ESM.pdf]

Article title: Plant-derived smoke water promotes rice root growth under phosphorus deficiency by modulating oxidative stress and photosynthetic gene expression

Journal name: Scientific Reports

Authors: Sompop Pinit, Lalichat Ariyakulkiat, and Juthamas Chaiwanon

Corresponding author affiliation: Center of Excellence in Environment and Plant Physiology, Department of Botany, Faculty of Science, Chulalongkorn University, Bangkok, Thailand

Corresponding author email: juthamas.c@chula.ac.th

SUPPORTING INFORMATION

Fig S1 Expression of Pi-starvation-induced (PSI) genes in the presence and absence of smoke water treatment.

Table S1 List of differentially expressed genes and expression levels (log_2fold change and p-values) (.xlsx)

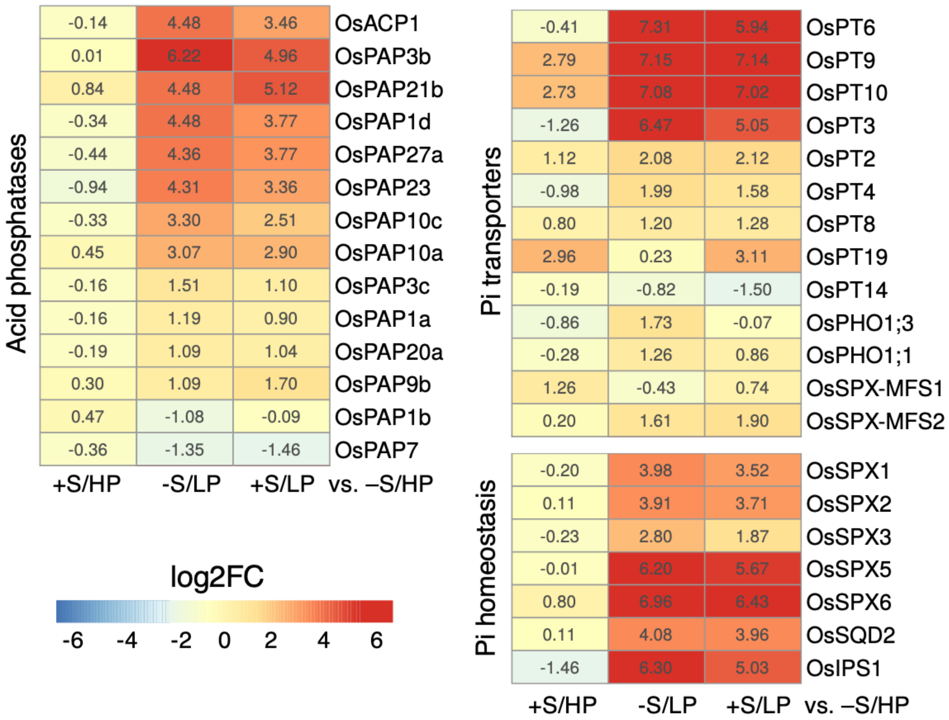

Supplemental Figure S1. Expression of Pi-starvation-induced (PSI) genes in the presence and absence of smoke water treatment.
Heatmaps represent log_2FC values of genes in the +S/HP, -S/LP or +S/LP compared with -S/HP conditions. Only genes that showed statistical significance in at least one of the comparisons were included in this figure.
